# Burden of Tick-Borne Encephalitis, Sweden

**DOI:** 10.3201/eid2802.204324

**Published:** 2022-02

**Authors:** Daniel Slunge, Anders Boman, Marie Studahl

**Affiliations:** University of Gothenburg, Gothenburg, Sweden (D. Slunge, A. Boman, M. Studahl);; Region Västra Götaland, Sahlgrenska University Hospital, Gothenburg (M. Studahl)

**Keywords:** Tick-borne encephalitis, hospitalization, mortality, sick leave, global burden of disease, disease cost, economic burden of disease, vector-borne infections, parasites, Sweden, meningitis/encephalitis, tick-borne encephalitis virus, TBEV

## Abstract

In recent decades, the incidence of tick-borne encephalitis (TBE) in Sweden has increased. To calculate the burden of disease over a 17-year period, we analyzed data from the Swedish National Health Data Register for TBE cases diagnosed during 1998–2014. We compared healthcare use and sick leave associated with 2,429 persons with TBE with a referent cohort of 7,287 persons without TBE. Patients with TBE were hospitalized for significantly more days during the first year after disease onset (11.5 vs. 1.1 days), logged more specialist outpatient visits (3.6 vs. 1.2 visits), and logged more sick leave days (66 vs. 10.7 days). These differences generally increased over time. The case-fatality rate for TBE was 1.1%. Our calculated cost of TBE to society provides a baseline for decisions on immunization programs. Analyzing register data, our study adds to clinical studies of smaller cohorts and model-based studies that calculate disease burden.

Tick-borne encephalitis virus (TBEV) is the cause of tick-borne encephalitis (TBE), an infectious disease of growing public health concern (*1*,*2*). In Sweden, the disease is caused by the European subtype (TBEV-Eu), which is transmitted by the vector tick *Ixodes ricinus* (*3*). Over the past 3 decades, the number of cases has dramatically increased, with an average of 391 notified cases annually during the past 5 years (2017–2021), corresponding to an incidence of 3.8 cases/100,000 population (*4*,*5*) (Figure 1). In certain regions of Sweden, however, the incidence among unvaccinated persons has been up to 8.5–12 cases/100,000 population (*6*). In Europe, only Lithuania, Latvia, Estonia, Czechia and Slovenia report higher notification rates, on national levels, than Sweden (*7*)*.* TBEV infection is mainly asymptomatic or associated with mild signs/symptoms (e.g., fever and general malaise) but may also cause neurologic signs/symptoms in persons in all age groups (*8*). Clinical studies show that children account for 10%–16% of TBE cases (*9*). Clinical presentation ranges from mild meningitis to severe manifestations such as meningoencephalomyelitis with a risk for respiratory insufficiency requiring ventilator support in an intensive care unit (ICU) (*10*–*12*). In Europe, ≈95% of case-patients with notified TBE require hospitalization (*13*).

**Figure 1:**
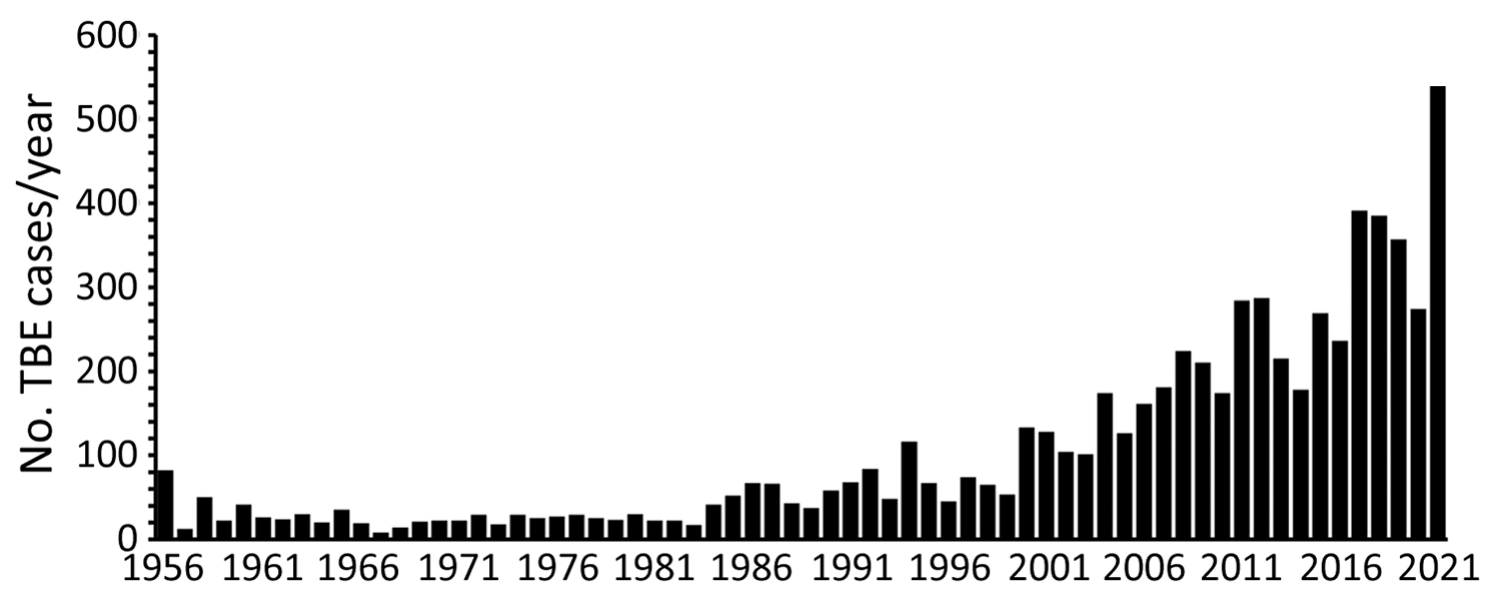
Reported tick-borne encephalitis cases per year, Sweden, 1956–2021. Tick-borne encephalitis became a notifiable disease in Sweden in July 2004; thus, the number of reported cases before 2005 is less certain than the number of cases from 2005 on. Source: Swedish Public Health Agency (https://www.folkhalsomyndigheten.se), 2021.

Although the case-fatality rate associated with TBEV-Eu (based on previous case series in Sweden) is estimated at only 0–1.4% (*14*,*15*), neurologic sequelae are common and often long lasting. The rate of incomplete recovery severely affecting quality of life at long-term follow-up is reported to be ≈40%–46% (*15*,*16*). There is no cure for TBE, but 2 inactivated TBEV-Eu vaccines resulting in 95%–100% immunogenicity are available (*17*).

In Europe, only Austria has implemented a national universal TBE vaccination program targeting the entire population, resulting in a pronounced decrease in TBE incidence (*18*,*19*). The growing incidence of TBE has stimulated discussion regarding the need for public vaccination programs in Sweden (*20*,*21*) and other countries in Europe (*22*–*24*), but thorough data concerning the burden of TBE are needed to determine cost-effectiveness.

Our purpose with this study was to provide a baseline concerning the burden of TBE to enable informed decisions on immunization programs and other healthcare interventions. We analyzed the overall burden of TBE in Sweden in terms of hospitalization, specialist outpatient visits, primary care visits and sick leave on the basis of register data on TBE case-patients and a matched cohort. For TBE case-patients, we also included the cost of death. The study was approved by the Regional Ethical Review Board in Gothenburg (no. 141-16).

## Materials and Methods

### Data Sources

**Figure 2:**
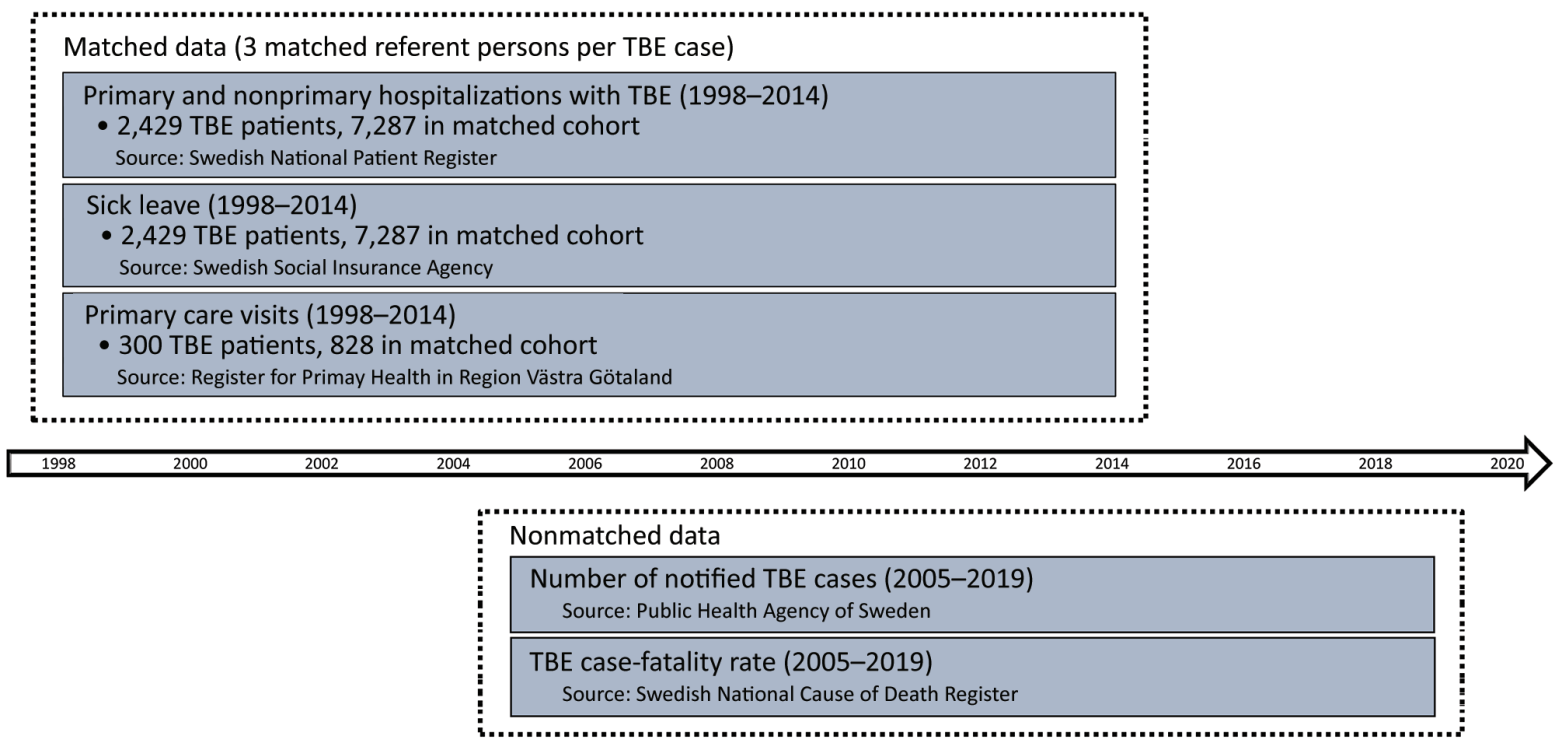
Sources and periods of matched and nonmatched data used in study of tick-borne encephalitis, Sweden. Swedish National Patient Register, https://www.socialstyrelsen.se; Swedish Social Insurance Agency, https://www.forsakringskassan.se; Register for Primary Health in Region Vastra Gotaland, https://www.vgregion.se; Public Health Agency of Sweden, https://www.folkhalsomyndigheten.se; Swedish National Cause of Death Register, https://www.socialstyrelsen.se.

We collected data from various sources and periods (Figure 2). We obtained data from the Swedish National Patient Register (provided by the National Board of Health and Welfare, https://www.socialstyrelsen.se) related to the diagnosis code for TBE (A84, International Classification of Diseases 10th revision [ICD-10]), including date of notification of TBE, among patients who received inpatient care or specialist outpatient care for this diagnosis during 1998–2014. This register includes patients for whom TBE was a primary cause for hospitalization and patients ill with TBE but for whom a different primary diagnosis was the cause for hospitalization. Including both primary and nonprimary diagnoses of TBE in the data ensures that no hospitalized TBE patients are omitted. Statistics Sweden (https://www.scb.se) created a matched referent cohort encompassing 3 referent persons per TBE case-patient, on the basis of sex, age in 2014, and county of residence.

The Swedish Social Insurance Agency (https://www.forsakringskassan.se) provided data concerning numbers of sick leave days and amount of sick leave compensation during the study period for the TBE case-patients and the referent cohort. By law, the Swedish social insurance system covers all residents 16–64 years of age and grants economic security when the ability to work is limited by >25% because of sickness, disability, or injury (*25*). Statistics Sweden provided the social security numbers of the referent cohort to the National Board of Health and Welfare, which provided the same information for the referent cohort as for the TBE case-patient group.

We obtained data from the Region Västra Götaland Primary Healthcare Register (https://www.vgregion.se) regarding primary care visits for persons with TBE and those in the matched cohort living in this region (1.7 million of 10 million inhabitants in Sweden). Data encompassed 5 years before through 5 years after TBE diagnosis.

TBE has been a notifiable disease in Sweden since July 1, 2004. The involved microbiology laboratories and the attending physicians are responsible for notifications to authorities. We obtained data on notified cases from the Public Health Agency of Sweden (https://www.folkhalsomyndigheten.se) for 2005–2019, including date of reported TBE diagnosis.

Data concerning death caused by TBE (ICD-10 code A84) were obtained from the Swedish National Cause of Death Register (https://www.socialstyrelsen.se), which covers ≈98% of deaths in Sweden. We calculated the case-fatality rate for 2005–2019, after TBE had become a notifiable disease in Sweden, and expressed it as a ratio between the number of deceased persons divided by the number of notified TBE cases in the register maintained by the Public Health Agency of Sweden.

### Data Analyses

We calculated TBE-related sick leave and healthcare consumption by analyzing the number of days of sick leave and hospitalization as well as primary care and specialist outpatient visits during years 1, 3, and 5 after TBE diagnosis, after which we compared the data with that from the referent cohort. By comparing these differences with the differences in healthcare use and sick leave days over the 3-year period before date of TBE onset, we segregated the effects exclusively caused by TBE from other potential differences between the TBE case-patients and the referent cohort. We used *t*-tests to determine whether differences between the TBE case-patients and the referent cohort were statistically significant. To account for potential sick leave days and healthcare visits resulting from TBEV infection before diagnosis, we defined the date of TBE onset as occurring 31 days before the TBE diagnosis (ICD-10 code A84) was made. Febrile TBEV-related illness precedes onset of encephalitis in the biphasic course of disease, which occurs in most patients. The duration of this febrile phase is usually ≈5 days (range 2–10 days), which is then followed by a symptom-free interval of ≈7 days (range 1–21 days) before onset of the actual TBE symptoms that prompt contact with either outpatient or inpatient care (*8*). Hence, we chose 31 days to encompass the maximum number of days of illness relating to TBE before diagnosis.

We established the date of diagnosis as the first date when a diagnosis of TBE (ICD-10 code A84) was made in either outpatient specialist or inpatient care. This date was chosen because there may be a delay of weeks to months before the Public Health Agency is notified after hospitalization and because the date on which the TBE diagnosis is entered in the Swedish National Patient Register usually corresponds to the date of hospital discharge.

We calculated the burden of TBE for each outcome from the differences in mean values between TBE case-patients and the referent cohort, while also taking into account differences in the baseline values 3 years before TBE onset. 

We calculated the cost of illness for all TBE patients in Sweden by using the following monetary values, based on the burden of disease estimates. The average cost per day of hospital stay during 2014–2018 was calculated to be €1,049, and the cost per specialist outpatient visit was €338, based on the cost per patient database from the Swedish Association of Local Authorities and Regions (*26*). The average cost per primary care visit was calculated to be €164, based on 2019 prices charged for a physician visit in Västra Götaland (*27*). The average cost per day of sick leave was calculated to be €199, based on loss of income and calculated by using the 2018 median monthly wage (€3,090) plus mandatory employer social security contributions in Sweden (36% of the wage), divided by the number of working days in that year (253 days) (*28*,*29*). For the purposes of this study, we counted 2 half days of sick leave as 1 full day. The cost of death caused by TBE was calculated to be €4.05 million, based on the value of a statistical life used by the Swedish Transport Administration (*30*). For all calculations, we used the following exchange rate: €1 equals 10 Swedish krona (SEK) (€1 is approximately equal to US $1.20). Statistical analyses were performed by using STATA version 16 (https://www.stata.com).

## Results

### TBE Diagnosis in the Swedish National Patient Register

Data obtained from the Swedish National Patient Register identified 2,429 reported patients hospitalized with TBE ICD-10 diagnosis code A84 during 1998–2014. Of these, 1,751 case-patients were entered in the register during 2005–2014. Over that same period, 2,047 TBE case-patients were reported in Sweden, indicating that 296 (14%) such case-patients did not require hospitalization. Of the 2,429 case-patients entered in the register, 995 (41%) were women and 1,434 (59%) men.

**Figure 3:**
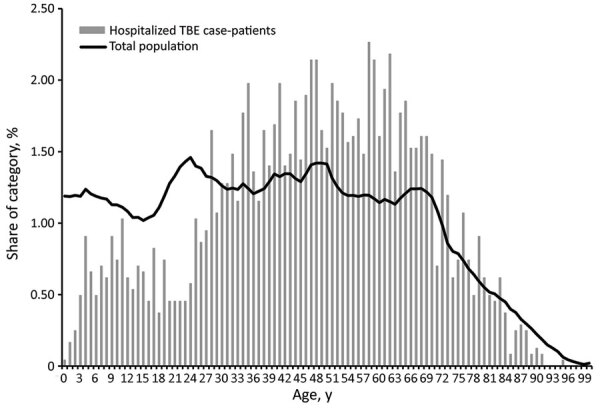
Percentage of hospitalized tick-borne encephalitis case-patients, by age, during 1998–2014 and percentage of population of Sweden in 2014, by age.

Mean age of the 2,429 TBE case-patients entered in the National Patient Register was 47 years (47.8 for women and 46.4 for men). Comparing the age distribution of TBE cases with the general population shows that hospitalization for TBE is skewed toward a higher mean age than the population at large (Figure 3).

### Death from TBE

A total of 39 TBE-related deaths were entered in the Swedish National Cause of Death Register during 2005–2019. During the same period, 3,681 TBE cases were reported to the Public Health Agency of Sweden, corresponding to a case-fatality rate of 1.1%. In all, 25 (64%) of the deceased patients were men and 14 (36%) were women; 35 (90%) of deceased patients were >60 years of age.

### Days of Inpatient Care and Number of Specialist Outpatient Visits

When we compared the burden of TBE in terms of healthcare use between TBE case-patients and the referent cohort at 3 years before and 1, 3 and 5 years after TBE onset date, we found that before TBE onset, differences in the average number of days spent in hospital care were small and statistically insignificant; however, after TBE onset, TBE case-patients spent significantly longer than the referent cohort in hospital care (Table 1). During the first year after TBE onset, case-patients were hospitalized an average of 11.5 days, compared with an average of 1.1 days for the referent cohort. These differences remained largely unchanged in the following years.

**Table 1:** Healthcare use and sick leave days for persons with TBE and the matched referent cohort, Sweden, 1998–2014*

Variable	Within 3 y before TBE	After TBE		
		Within 1 y	Within 3 y	Within 5 y
Days hospitalized				
Case cohort, mean (no.)	1.35 (2,228)	11.50 (2,274)	12.73 (1,863)	14.69 (1,466)
Referent cohort, mean (no.)	2.07 (6,684)	1.12 (6,822)	3.27 (5,589)	5.64 (4,404)
Difference in means	0.28	10.38†	9.46†	9.05†
Specialist outpatient visits				
Case cohort, mean (no.)	3.92 (2,228)	3.65 (2,270)	6.78 (1,863)	9.45 (1,466)
Referent cohort, mean (no.)	3.31 (6,684)	1.22 (6,810)	3.53 (5,583)	5.74 (4,398)
Difference in means	0.62†	2.42†	3.25†	3.71†
Days of sick leave				
Case cohort, mean (no.)	63.3 (1406)	66.0 (1,434)	122.6 (1,169)	144.0 (902)
Referent cohort, mean (no.)	51.4 (2765)	10.7 (2,802)	45.9 (2,286)	78.1 (1,793)
Difference in means	11.82‡	55.32†	76.66†	65.85†
Primary healthcare visits				
Case cohort, mean (no.)	9.19 (280)	6.44 (276)	14.43 (228)	23.29 (175)
Referent cohort, mean (no.)	10.55 (779)	3.90 (763)	11.54 (638)	19.55 (502)
Difference in means	−1.36	2.54†	2.89§	3.74

*TBE, tick-borne encephalitis. †Significance at the 1% level. ‡Significance at the 5% level. §Significance at the 10% level.

Within the 3-year period before date of TBE onset, the average number of specialist outpatient visits was slightly higher among patients with a TBE diagnosis than among the referent cohort (Table 1). By 1 year after date of onset of TBE, these differences became much more pronounced and grew over time; after 5 years, the average difference was almost 4 visits.

### Sick Leave Days

Compared with the referent cohort, patients with TBE spent an average of 12 days more on sick leave over the 3-year period before TBE onset (Table 1). One year after TBE onset, this difference increased significantly; those with TBE spent an average of 66 days on sick leave, compared with 11 days for the referent cohort. Three years after TBE onset, this difference was even greater but decreased again; after 5 years, it returned to the same level as 1 year after the onset of TBE.

### Primary Care Visits

The number of primary care visits during the 3 years before TBE onset did not differ significantly between the 2 groups (Table 1). However, in the first year after TBE onset, the number of visits was substantially higher for those with a TBE diagnosis. This difference declined over time, and after 3 years there were no statistically significant differences.

### Burden of TBE and Cost of Illness

Calculations of the burden of TBE in terms of healthcare use and sick leave and the associated cost of illness per TBE case 1, 3 and 5 years after TBE onset take into account the differences between the TBE case-patients and referent cohort within the 3-year period before date of TBE onset (Table 2). The average cost of illness for 1 TBE case-patient was ≈€20,504 during the first year after TBE onset. Days spent in hospital accounted for 52% of this cost; days on sick leave, 42%. Specialist and primary care visits accounted for 3% each. The cost grew by ≈€3,600 in years 2 and 3 to a cumulative cost of €24,126 by 3 years after TBE onset. During years 4–5, the per patient cost decreased by about €2,300 to a cumulative cost of €21,834/TBE case 5 years after TBE onset. Over time, the share of costs for inpatient care decreased to 41%, and the costs associated with sick leave increased to 49% at 5 years after TBE onset. The share of costs for specialist visits increased only slightly to 5% and for primary care visits to 4%.

**Table 2:** Burden of TBE in terms of healthcare use, sick leave days, and cost of illness per case, Sweden, 1998–2014*

Variable	Within 1 y after TBE onset				Within 3 y after TBE onset				Within 5 y after TBE onset		
Healthcare use and sick leave, no.	Cost of illness/ case, €	Share of total cost of illness, %	Healthcare use and sick leave, no.	Cost of illness/ case, €	Share of total cost of illness, %	Healthcare use and sick leave, no.	Cost of illness/ case, €	Share of total cost of illness, %
Days hospitalized	10.10	10,599	51.7		9.19	9,638	39.9		8.77	9,203	41.2
Specialist outpatient visits	1.80	610	3.0		2.63	890	3.7		3.09	1,045	4.8
Primary care visits	3.90	639	3.1		4.25	696	2.9		5.10	836	3.8
Days of sick leave	43.50	8,656	42.2		64.80	12,902	53.5		54.00	10,750	49.2
Total	NA	20,504	NA		NA	24,126	NA		NA	21,834	NA

*Calculations account for differences between TBE case-patients and referent cohort during a 3-y period before date of disease onset. NA, not applicable; TBE, tick-borne encephalitis.

Of the 359 TBE cases registered in Sweden in 2019, a total of 4 case-patients died of this disease, equating to a cost of illness of €7.3 million and a cost of death of €16.2 million, for a total cost of €23.5 million (Table 3). The corresponding average annual cost for 2015–2019 is €24.5 million; the cost of illness accounts for €6.6 million and that of death €17.8 million.

**Table 3:** Cost of illness and death from TBE in Sweden in 2019 and per year 2015–2019*

Variable	2019	2015–2019, average yearly cost
Registered TBE cases, no.	359	1,641
Deaths caused by TBE, no.	4	22
Cost of illness, €	7,279,054†	6,639,317†
Cost of death, €	16,200,000 ††	17,820,000 ††
Total cost of illness and death, €	23,479,054	24,459,317

*TBE, tick-borne encephalitis. †No. registered TBE cases minus the number of deaths caused by TBE times the cost of illness per TBE case within 1 y after TBE onset (€20,504). ††No. deaths caused by TBE times the value of a statistical life in Sweden (€4.05 million).

## Discussion

The burden of tick-borne encephalitis was higher than previously estimated. This study, based on register data in Sweden, where underreporting of TBE is demonstrably low (*31*), shows that TBE poses a substantial burden as measured by use of healthcare and sick leave. 

The average of 11.5 days of hospitalization during the first year after TBE onset found in this study is similar to the 12 days found in a register-based study in Latvia, which covered ≈2,000 TBE cases (*32*). Our figures fall between findings of smaller studies from Slovenia (9 days) (*33*) and Germany (18 days) (*10*). By comparison, an earlier study in Sweden found that herpes simplex encephalitis, one of the most severe viral encephalitides, required an average of 55 days of hospitalization (*34*). Not surprisingly, the same pattern was observed in a recent US study quantifying the health economic effects of viral encephalitis, which found that patients with herpes simplex encephalitis were associated with longer cumulative hospital stays than were patients with all other viral encephalitides (*35*). However, comparisons between TBE and other viral encephalitides are complicated by differences in severity and prognosis.

According to our analysis, hospitalization accounted for only about half of the disease burden from TBE but sick leave days accounted for a substantial share. We found a difference in sick leave days taken before the onset of TBE, and those who received a TBE diagnosis took more sick leave days on average. We see no obvious explanation for this difference. Among those with TBE, sick leave days increased sharply over the first 3 years after TBE onset. In the 4–5 years after TBE onset, patients with this illness instead took fewer sick leave days on average than the referent cohort. This finding may be associated with rules regarding the maximum number of sick leave days allowed, but the register data on which this study was based did not permit further analysis.

This pattern of sick leave for TBE differs somewhat from that for another tickborne disease, neuroborreliosis, which was investigated through a register study in Denmark that showed that more days were taken for sick leave during the first year but tapered rapidly thereafter (*36*). In that study, 2 years after diagnosis the number of sick leave days did not differ substantially between neuroborreliosis case-patients and controls. The differences in sick leave pattern between these 2 diseases probably reflect the moderate to severe sequelae of TBE in up to one half of case-patients at long-term follow-up, compared with neuroborreliosis, for which only 12% experienced sequelae that affected their activities of daily living (*15*,*16*,*37*).

The TBE case-fatality rate calculated in this study (1.1%), based on register data from a 15-year period, is considerably higher than that reported for Sweden during the historical period 1956–1989 (0.5%) (*38*) and among the average notified cases from 23 countries in Europe during 2012–2016 (0.5%) (*13*). However, in smaller cohorts from different parts of Europe, fatality rates vary from 0.75% to 3.6% (*16*,*39*). Fatality rates may vary according to several factors, including virus virulence, sensitivities of different surveillance systems, variations in how death is recorded in different countries, demographics (e.g., age), and immunosuppression; the latter 2 factors are known to increase severity and death (*39*–*43*).

As in previous studies (*10*,*16*,*22*), we found that TBE affects more men than women, probably because compared with men, women are more likely to use protective measures, appreciate the risk, and be more knowledgeable about tickborne diseases (*44*–*46*). Moreover, the mortality rate was somewhat higher among men than women.

Translating the societal burden of TBE that arises from increased healthcare use and sick leave into monetary cost of illness is helpful for assessing the cost-effectiveness of immunization programs and other healthcare interventions. The average cost of hospitalization and specialist outpatient visits during the first year after TBE diagnosis, derived from this study, is of the same order of magnitude as earlier estimates used in models to calculate the cost-effectiveness of TBE immunization programs (*21*). However, we found that it is also essential to include the substantial cost of illness related to sick leave when comparing costs and benefits of TBE immunization programs. Excluding sick leave-related costs from such analysis would underestimate the cost of illness, especially after the year of incidence, because the percentage of costs associated with sick leave increases over time.

Using a referent cohort comparison in this study made it possible to identify the net burden of disease through analysis of the differences between the 2 groups before and after TBE onset. Including the costs of healthcare use and sick leave of only the TBE case-patients would overestimate the cost of illness.

The proportion of TBE case-patients requiring intensive care could not be reliably identified from the registers, which poses a study limitation. However, ICU stays are probably associated with a large part of hospitalization costs, as shown in a large US study of >25,000 adult patients with meningitis and encephalitis (*47*). One of few studies to include the need for intensive care over the course of TBE showed that 12% of 656 TBE patients in Germany received treatment in an ICU for an average of 12 days (*10*). Another study of 448 TBE patients in Slovenia showed that 7% received treatment in ICUs (*33*). Assuming that 10% of the TBE patients in our study received treatment in an ICU for an average of 12 days, at an average cost of €6,500 per day (*48*), the estimated cost of hospitalization 1 year after TBE onset would increase by 62% to €17,140 per case and the total cost of illness for the 359 TBE cases in Sweden in 2019 by 32% to €9.6 million.

Another study limitation is that our burden of disease calculations did not take into account less-tangible costs, such as the pain and suffering associated with long-term sequelae commonly associated with TBE or changes in recreational behavior motivated by the increased risk for TBE (*49*). The short time perspective of the study is also a limitation because it only analyzes healthcare use and sick leave for 5 years after TBE onset. Some studies do address long-term effects (*50*), but these need to be complemented by additional studies that take into account the costs associated with the long-term sequelae of TBE.

By analyzing data from the Swedish National Patient Register, our findings add to clinical studies of smaller cohorts (*10*,*15*) and to model-based studies that calculate disease burden (*21*–*23*). Such studies are of value to patient care with regard to detecting cognitive and neurologic impairments, and they provide an estimate of the frequency, nature, and severity of sequelae. Register data relating to healthcare use, sick leave, and death provide a broader base of knowledge concerning the burden of TBE. The calculated cost of illness per TBE case in this study provides a baseline for analyses of cost-effectiveness of immunization programs, which frequently rely on cost data from other diseases to estimate costs for hospitalization and specialist outpatient visits in subsequent years.
